# Simultaneous Identification on Tomato Variety and Maturity Based on Local and Global Feature Fusion

**DOI:** 10.3390/s25237313

**Published:** 2025-12-01

**Authors:** Shaohuang Bian, Jun Zhou, Qinxiu Gao, Chengxi Yi, Wenzhuo Chen, Feng Huang

**Affiliations:** 1 College of Information and Electronic Engineering, China Agricultural University, Beijing 100083, China; shaohuangbian@cau.edu.cn (S.B.); sy20243082029@cau.edu.cn (J.Z.); sy20233081884@cau.edu.cn (Q.G.); ycxself@cau.edu.cn (C.Y.); wenzhuochen@ncist.edu.cn (W.C.); 2 College of Science, China Agricultural University, Beijing 100083, China

**Keywords:** YOLOv8n, simultaneous identification, tomato variety and maturity, local and global feature fusion

## Abstract

Varieties show their unique characteristics in morphology, growth, and fruits. Tomato maturity is related to multiple dimensional characteristics including color, texture, smell, etc. An effective classification method of tomato variety and maturity is crucial for evaluating its growth and yield. However, due to the complex growth environment, some problems such as leaf occlusion and fruit shaded by each other make it difficult to accurately and efficiently identify them. To solve these problems, this study innovatively proposes a simultaneous detection model on tomato variety and maturity based on improved YOLOv8n, with the combination of frequency-adaptive dilated convolution (FADC) feature extraction module and the high-level screening-feature path aggregation network (HSPAN) with the aim of local and global feature fusion by the channel attention module and feature selection fusion mechanism. In addition, we use the Powerful-IoU (PIoU) loss function to replace the original Complete IoU (CIoU) to enhance the accuracy of bounding boxes. We also introduce a dynamic detection head as the final output of the model, which can adaptively adjust the focus of feature extraction according to the color and size of tomato fruits, thereby improving the recognition accuracy. Experimental results show that our model with better global perception capability achieves the highest detection accuracy and lower computation complexity among the comparative models.

## 1. Introduction

Tomato is an essential vegetable that is widely grown and consumed around the world. Tomato is a common ingredient in daily diet and plays a crucial role in agriculture and economy. As one of their vegetables, tomato has become an important choice for farmers to increase their income due to their easy-to-grow and high yield. Till now, tomato maturity judgement had mainly relied on manual methods. Although manual methods are sometimes effective, there are some problems such as solid subjectivity, prolonged time consumption with fruit waste, and high labor cost [1]. In addition, external environmental factors such as light and temperature can also affect the judgement results. Thus, exploring more efficient and accurate recognition methods is imperative [2].

This article proposes a tomato variety and maturity synchronous detection framework based on an improved YOLOv8n. The main innovations are as follows. (1) Introduce an adaptive convolution module to enhance the ability to extract fruit features at different scales. (2) Design a feature pyramid structure for high-level screening and multi-scale fusion to achieve explicit fusion of local and global features. (3) Using bounding box regression loss (PIoU) can achieve more accurate bounding box regression. (4) Introduce a dynamic detection head that combines scale, spatial, and task aware attention, allowing for explicit modeling of fruit size and spatial position. Through these improvements, the model can maintain high detection accuracy and low computational cost even in scenes with severe occlusion, complex lighting, and mixed varieties.

The rest of this article is organized as follows. Section 2 provides a comprehensive analysis and review of relevant research on tomato variety and maturity identification. Section 3 introduces the proposed method. Section 4 introduces the experimental results and discussion. Section 5 summarizes the research findings and conclusions.

## 2. Related Work

Since 2017, with the breakthrough of deep learning in image classification and object detection, research on tomato detection has shifted from traditional color thresholding methods to data-driven convolutional neural network (CNN) frameworks. Early work mainly used two-stage detection method (for example, “locating fruits first and then determining maturity”) based on convolutional neural networks (CNN) and its improvement (including VGG and DenseNet, and Faster R-CNN and Mask R-CNN) [3,4,5,6,7,8,9,10]. Sun et al. [6] proposed a tomato organ recognition method based on CNN with feature pyramid, in which detailed underlying features and high-level semantic features were fused by multi-scale feature fusion. Xu et al. [11] proposed a tomato recognition and location model based on Mask R-CNN with the dual-mode data fusion of RGB and depth images. These two-stage methods significantly improve recognition accuracy compared to traditional image processing, but generally have high computational costs and low computation speed and the accuracy needs to be enhanced further. In addition, most of the research in this stage focuses on a single task, lacking the ability to jointly identify variety and maturity [12].

The development of deep learning, one-stage detection algorithms with faster computation speed is gradually becoming mainstream [13]. For example, researchers used YOLOv5 with feature pyramids and attention mechanisms to enhance small object detection capabilities [14,15] or used an anchor-free detector model with an added attention mechanism to the backbone part and introduced circular representation for optimizing the detector to improve the feature expression ability [16]. In this stage, the balance between speed and accuracy is a significant improvement compared to those single-stage detection methods. However, most of the research in this stage mainly only focuses on single tasks and rarely involves multi-task detection. Meanwhile, the computation complexity still needs to be further decreased for deployment in practical application devices.

At present, the research focus is gradually shifting towards “balancing accuracy, robustness, and lightweight deployment” by one-stage algorithms. YOLOv8 and its later version are the representative models. For example, the multi head self-attention mechanism or locally sensitive large kernel attention mechanism was introduced to YOLOv8 to improve tomato maturity grading and counting in complex environments [17,18], and the proposed GFS-YOLO11 can remain high performance in multi-variety tomato recognition [19]. However, most of these methods still remain at the level of local feature enhancement or simple feature concatenation, lacking high-level semantic filtering and global local fusion mechanisms [20,21]. Meanwhile, the insufficient utilization of fruit size and spatial location information limits the generalization performance in complex scenarios. On the other hand, for the deployment in practical edge devices, some studies have attempted to reduce computational overhead through pruning, quantization, and lightweight convolution [22,23].

In summary, the existing methods still face several challenges in tomato detection. Firstly, there is a lack of lightweight models that can simultaneously detection variety and maturity; secondly, the feature fusion mechanisms are mostly shallow concatenation or weighting, which makes it difficult to balance global semantics and local details; thirdly, the bounding box size and spatial position of tomatoes have not been fully utilized, and these are particularly crucial for improving detection accuracy; and fourthly, high-precision methods have excessive computational complexity, while lightweight methods often sacrifice recognition performance and have not yet achieved an ideal balance between accuracy and efficiency.

## 3. Materials and Methods

### 3.1. Dataset

**Figure 1:**
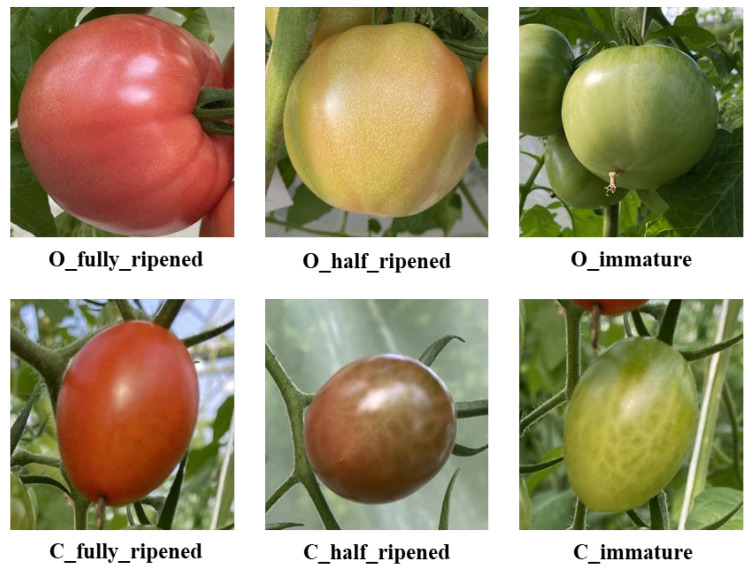
Data samples (O represents ordinary tomatoes and C represents cherry tomatoes).

The constructed dataset in this study is based on the Laboro Tomato open-access dataset [24], which is an image dataset of tomatoes with different maturity collected in a greenhouse in winter (15 December 2019). Our constructed dataset included two varieties of tomatoes (ordinary tomatoes with big, flat round fruits and cherry tomatoes with small, long round fruits), and each variety has three categories according to their maturity degree, including fully ripe (all red), half-ripe (red white or red green), and immature (completely green) shown in Figure 1.

**Figure 2:**
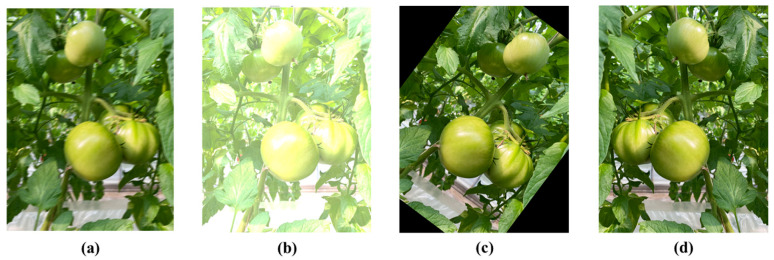
Image enhancement examples: (**a**) blurring processing, (**b**) brightness enhancement, (**c**) rotation, and (**d**) flipping.

In order to generate more diversified tomato images and improve the generalization of the model, data augmentation operations were performed on our dataset, including blurring, brightness enhancement, rotation, and horizontal flipping operations, shown in Figure 2. The enhanced dataset was divided into training sets (800 images), validation sets (100 images), and test sets (100 images) according to the ratio of 8:1:1.

### 3.2. The Proposed Improved YOLOv8n

**Figure 3:**
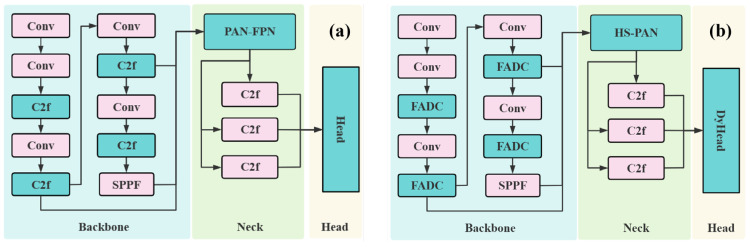
(**a**) The basic YOLOv8n model and (**b**) the improved model structure.

This study proposes a tomato variety category and maturity detection model based on the YOLOv8n. The basic and improved YOLOv8n are shown in Figure 3. The basic model mainly consists of three parts: backbone, neck, and head. The backbone extracts the features from the input image and outputs the feature maps in different sizes. The neck part is for feature fusion, and the head is for feature detection and result output. In the backbone of the improved YOLOv8n, the proposed Frequency-Adaptive Dilated Convolution (FADC) module was used to replace the original C2f module. In the neck part, the proposed high-level screening-feature path aggregation network (HS-PAN) replaced the original PAN-FPN. In the head part, the dynamic head (DyHead) replaced the original head.

The improvements in Figure 3b mainly include the following aspects: (1) the proposed FADC module can effectively capture features of different scales by dynamically adjusting the dilation rate of the convolution kernel, thereby improving the model’s recognition performance especially in complex scenarios including different varieties and different maturity of tomatoes; (2) in order to fuse features of different sizes and remove redundant information while retaining important feature information, the HS-PAN module was proposed, which uses channel attention and SFF modules to enhance the model’s ability to fuse feature information of different scales; (3) different tomatoes may obscure in shape and size, so accurate bounding boxes can help the model better distinguish different types. The PIoU loss function was adopted, focusing on the positioning accuracy of the bounding box and considering the association between the bounding box and the category so that the model can adaptively optimize the prediction of the box and improve the classification performance; and (4) the dynamic detection head with attention mechanism for feature detection can adaptively adjust the focus of feature extraction based on task perception, spatial perception, and scale perception, enhance the ability to distinguish different varieties of tomatoes and their maturity degree, and significantly improve the recognition accuracy of tomatoes.

#### 3.2.1. Adopting Frequency-Adaptive Dilated Convolution (FADC) Module in Backbone

**Figure 4:**
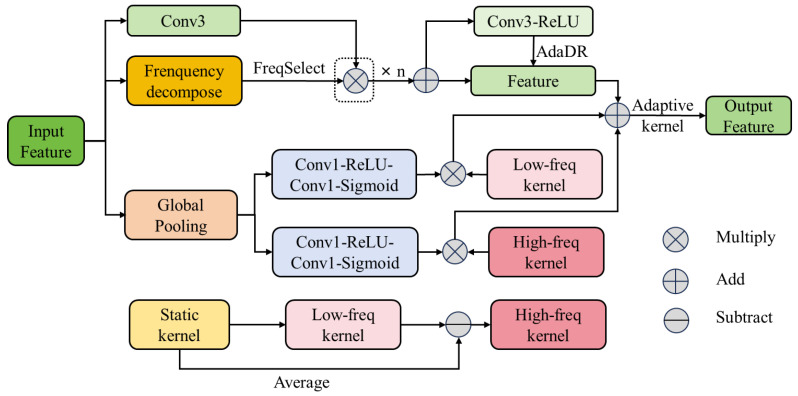
Frequency-adaptive dilated convolution (FADC) module.

The frequency-Adaptive Dilated Convolution (FADC) module (shown in Figure 4) consists of three important modules: adaptive dilation rate (AdaDR), adaptive kernel (AdaKern), and frequency selection (FreqSelect) [25]. First, the feature map X was input into the frequency decomposition module to generate multiple frequency channels $X_{freq},$ then to the frequency selection module for selecting frequency. Second, the model calculates the corresponding dilation rate $\hat{D}\left(p\right)$ for each pixel *p* in the feature map according to the strategy learned during training and maps it to a new feature map where each pixel corresponds to a dilation rate. In addition, the weight of the adaptive kernel is decomposed into low-frequency and high-frequency parts, and the ratio of these two parts is dynamically adjusted according to the input features. The formula is as follows:

(1) $$W^{\prime }=\lambda_{l}\cdot \bar{W}+\lambda_{h}\cdot \hat{W}$$

here, $W^{\prime }$ represents the dynamically adjusted weight, $\lambda_{l}\;\mathrm{and}$ $\lambda_{h}$ represent the weights of the low-frequency and high-frequency component, respectively, and $\bar{W}$ and $\hat{W}$ represent the average value of the convolution kernel (the low-frequency component) and the residual part (the high-frequency component), respectively. Finally, the adaptive dilation rate is used for convolution operation, and the formula is as follows:

(2) $$Y\left(p\right)=\sum_{i=1}^{K\times K}W_{i}\cdot X_{freq}\left(p+∆p_{i}\times \hat{D}\left(p\right)\right)$$

here, $Y\left(p\right)$ is the pixel value of position p in the output feature map of the adaptive dilated convolution, $W_{i}$ represents the ith weight in the convolution kernel, and $∆p_{i}$ represents the offset of each element in the convolution kernel. To find the optimal $\hat{D}\left(p\right)$, the optimization function of the adaptive dilation rate was used as follows:

(3) $$\theta =\underset{\theta }{\max}\left(\sum_{p\in {HP}^{-}}\hat{D}\left(p\right)-\sum_{p\in {HP}^{+}}\hat{D}\left(p\right)\right)$$

here, θ represents the optimization parameter, ${HP}^{-}$ represents a set of pixels with lower high-frequency components, and ${HP}^{+}$ represents a set with higher high-frequency components. For low-frequency areas (${HP}^{-}$), such as the outline and shape of tomatoes and background information, a more significant dilation rate was used to capture more contextual information and increase its receptive field, which helps to determine the location of tomatoes in the scene and their essential appearance characteristics. For high-frequency areas (${HP}^{+}$), such as tomatoes with higher maturity, the color usually changes significantly, so a lower dilation rate was used to ensure that high-frequency details are not lost, thereby preserving adequate feature information. In addition, the obtained high-frequency features can help identify small objects or partially occluded small tomatoes and enhance the detection ability of tomatoes. In the YOLOv8n model structure, the C2f module in the backbone was replaced by the FADC module to realize the feature extraction of different frequencies.

#### 3.2.2. Improvements on PANet Structure in Neck

The YOLOv8n neck part adopts the PANet (path aggregation network) structure, which is a neural network model for object detection with the aim to solve the detection problems of multi-scale objects and complex scenes [26]. PANet contains a top-down path aggregation module and a bottom-up path aggregation module. In the top-down path aggregation module, information is gradually transferred from high-level features to low-level features. In each level, high-level features are horizontally connected, and point-by-point, they are added with low-level features to fuse the information of different scales. In the bottom-up path aggregation module, information is gradually transferred from low-level features to high-level features. The low-level features are fused with high-level features after down-sampling. Through this design, PANet can integrate feature information of different scales.

**Figure 5:**
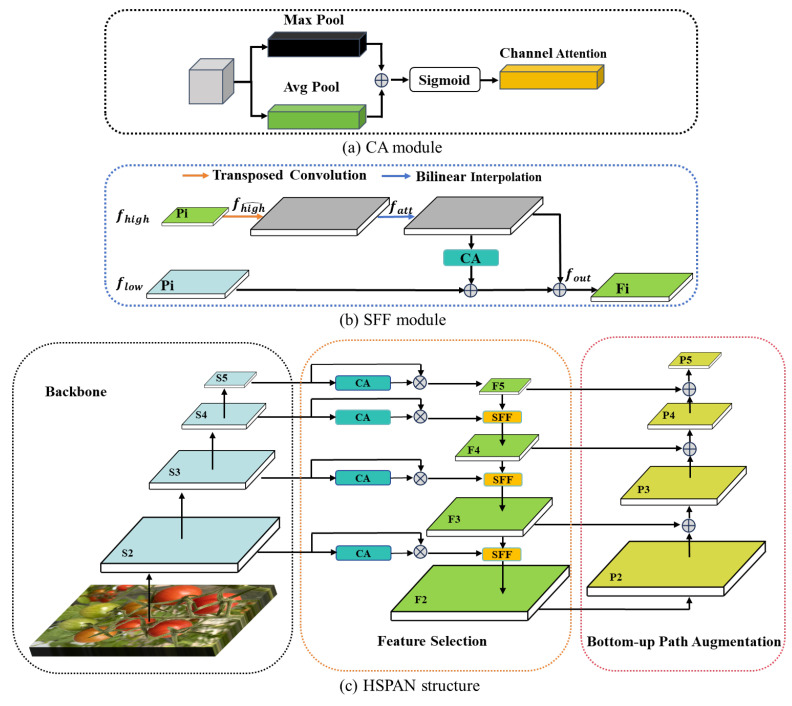
Improved high-level screening-feature path aggregation network.

In the detection and classification of tomato variety and maturity, the data features are relatively wealthy, including the information such as size, color, brightness, and texture. Many features of different sizes are input into the network structure, which makes the fusion process more complicated. In addition, features of different sizes may contain repeated or redundant information, which will affect the detection accuracy and performance of the model. Therefore, we add the CA and SSF modules (shown in Figure 5) to the low-level and high-level feature fusion stages in PANet structure to integrate features from different network layers to make full use of multi-scale information [27]. The SFF module enhances the ability to capture details and contextual information by fusing low-level and high-level features. The CA module adaptively adjusts the weights of feature channels so that the model can focus on more critical feature channels while suppressing irrelevant information. The calculation formula of CA and SFF are as follows:

(4) $$f_{CA}=Sigmoid\left(Pooling\left(f_{in}\right)\right)$$

here, Pooling includes average pooling and maximum pooling. The input feature is $f_{in}\in R^{C\times H\times W},$ with *C* being the number of channels, and *H* and *W* being the height and width of the feature map, respectively.

The SFF module mainly processes high-level features, filters low-level features, and then fuses the two to enhance the feature expression ability of the model and improve the detection performance. The formula is as follows:

(5) $$\left\{\begin{array}{c} f_{att}=BL(T\_Conv(f_{high}))\\ f_{out}=f_{low}*CA\left(f_{att}\right)+f_{att} \end{array}\right.$$

here, $f_{high}$ and $f_{low}$ represent high-level and low-level features, T_Conv represents transposed convolution, which is used to expand the size of the high-level feature map, and BL represents bilinear interpolation, which is used to align the size of the high-level feature map with the low-level feature map. The processed high-level feature map $f_{att}$ is consistent with the low-level feature map. We multiply $f_{att}$ with the attention weight of the high-level feature map (generated by Formula (9)), weight the low-level features, and then add them to the high-level features $f_{att}$ to obtain the final high-level and low-level fusion information $f_{out}$.

Based on CA and SFF modules, the high-level screening-feature path aggregation network (HSPAN) was proposed, which consists of three main parts (backbone, feature selection, and bottom-up path augmentation), shown in Figure 5c. First, the backbone extracts multi-level features of the input image layer by layer, where shallow high-resolution features (S2, S3) correspond to local details such as skin texture and color gradient, while deep low-resolution features (S5, S6) correspond to global context such as fruit color distribution and maturity semantics. In the feature selection stage, CA first performs adaptive recalibration on local channels to suppress redundancy caused by lighting and background noise. SFF then performs cross scale fusion between the recalibrated local details and the up-sampled global semantics, achieving explicit “local global feature fusion”. Finally, the bottom-up path enhancement returns the fused features through clear horizontal connections, further enhancing the precise positioning information within the global semantic framework, thereby improving the detection accuracy of tomato variety and maturity without increasing computational complexity.

#### 3.2.3. Improvement of IoU Loss Function

YOLOv8n uses CIoU as the intersection over the union loss function. CIoU is an extension of the traditional IoU (Intersection over Union), which implements bounding box regression by considering the overlapping area, center point distance, and aspect ratio simultaneously. However, due to the small overlapping area of cherry tomatoes, the loss calculation needs to be more sensitive in the actual tomato detection task. It may not be able to effectively capture the characteristics of cherry tomatoes, resulting in poor performance of CIoU in the detection task. In addition, the background of the collected tomato dataset is usually complex, which makes CIoU unable to accurately evaluate the boundaries of the object, thereby affecting the classification accuracy of maturity, especially in the case of severe background interference.

**Figure 6:**
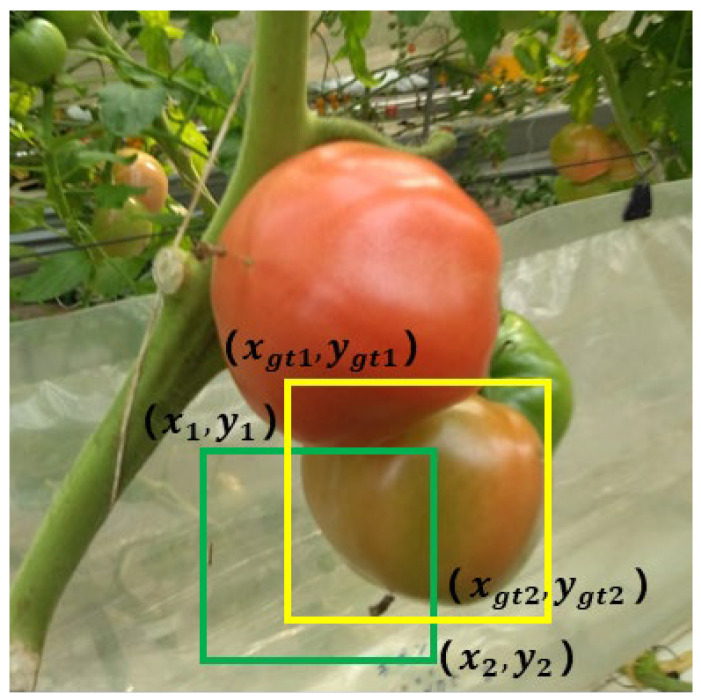
The parameter diagram of PIoU. Green and yellow squares represent the predicted box and the true box, respectively.

Therefore, we use the gradient adjustment function PIoU [28] that combines the object size and the quality of the anchor box. PIoU introduces an adaptive penalty factor *P* to adjust the loss function using the difference in coordinates between the diagonal vertices of the actual box and the predicted box so that the predicted box can approach the actual box faster, as shown in Figure 6. The coordinates of the upper left and lower right corner of the actual box (i.e., ground truth) are ($x_{gt1},y_{gt1}$) and ($x_{gt2},y_{gt2}$), and their predicted box are ($x_{1},y_{1}$) and ($x_{2},y_{2}$), respectively. The loss function is calculated as follows:

(6) $$\left\{\begin{array}{c} P=(\frac{d_{w1}}{w_{gt}}+\frac{d_{w2}}{w_{gt}}+\frac{d_{h1}}{h_{gt}}+\frac{d_{h2}}{h_{gt}})/4\\ f\left(P\right)=1-e^{-P^{2}}\\ L_{PIoU}=1-IoU+f\left(P\right) \end{array}\right.$$

in which $d_{w1}=x_{gt1}-x_{1}$, $d_{w2}=x_{gt2}-x_{2}$, $d_{h1}=y_{gt1}-y_{1}$, $d_{h2}=y_{gt2}-y_{2}$, ${w_{gt}=x}_{gt2}-x_{gt1}$, $h_{gt}=y_{gt1}-y_{gt2}$, and *f*(*P*) are the gradient adjustment functions based on the quality of the anchor box. Compared with CIoU used by YOLOv8n, PIoU can effectively guide the anchor box to regress more directly toward tomato size in the image. This mechanism improves the ability to focus on medium-quality anchor boxes, allowing the model to better utilize high-quality samples for learning, thereby improving the positioning accuracy, especially in the case of complex backgrounds and overlapping tomato fruits.

#### 3.2.4. Improvements to the Detection Head

The original detection head of YOLOv8n is the core of its object detection and is responsible for generating the final detection results. However, in actual tomato detection, the characteristics of different tomato maturity and sizes vary greatly. For example, the color of tomatoes with lower maturity may be similar to the background color from green leaves, and the sizes of different tomatoes are different. In addition, due to different photo shooting angle, actually similar tomatoes may present differ feature information. The detection head will be more sensitive to information such as the size and spatial position of tomatoes and can also perform multi-category detection.

**Figure 7:**
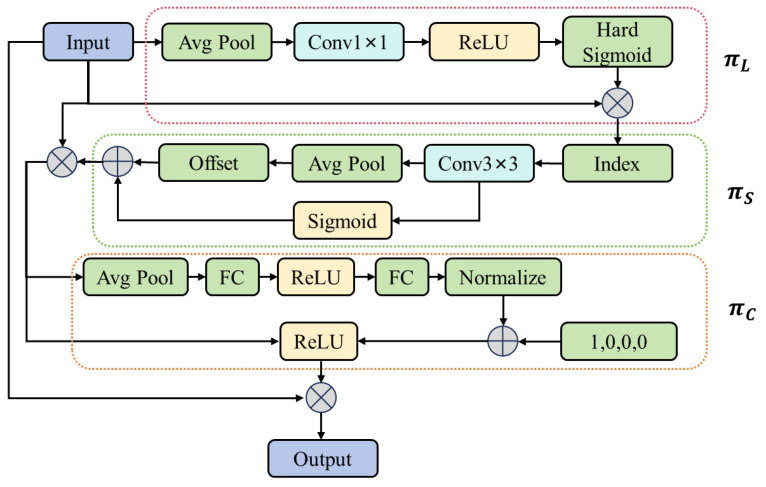
DyHead structure.

Therefore, in this study, we use a dynamic head [29] with the structure shown in Figure 7 to generate the final detection results. The dynamic detection head combines the attention mechanism with the object detection head to perform scale-awareness between feature levels and dynamically integrate the importance of different feature layers. It performs spatial-awareness between spatial positions, pays attention to the important space in the feature map, perform task-awareness between output channels, and adjust the activation of feature channels according to different tasks.

The formula for dynamic self-attention is as follows:

(7) $$W\left(F\right)=\pi \left(F\right)\cdot F$$

here, $\pi \left(F\right)$ is the attention function and F is the input tensor. They decompose the attention mechanism into scale attention (for scale-aware), spatial attention (for spatial-aware) and task attention (for task-aware) as follows:

(8) $$W\left(F\right)=\pi_{C}\left(\pi_{S}\left(\pi_{L}\left(F\right)\cdot F\right)\cdot F\right)\cdot F$$

here, $\pi_{L}\left(F\right)$ is scale attention, $\pi_{S}\left(F\right)$ is spatial attention, and $\pi_{C}\left(F\right)$ is task attention. They are expressed in Equations (9)–(11), respectively.

(9) $$\pi_{L}\left(F\right)\cdot F=\sigma \left(f\left(\frac{1}{SC}\sum_{S,C}F\right)\right)\cdot F$$

here, S represents the spatial dimension, C represents the channel dimension, $f\left(\cdot \right)$ is a linear function implemented through a 1 × 1 convolution layer, and $\sigma \left(x\right)$ is the sigmoid function.

(10) $$\pi_{S}\left(F\right)\cdot F=\frac{1}{L}\sum_{l=1}^{L}\sum_{k=1}^{K}w_{l,k}\cdot F_{l}\left(p_{k}+∆p_{k};c\right)\cdot ∆m_{k}$$

In Equation (10), L represents the number of feature layers, and K represents the number of those sparsely sampled. The outer summation aggregates responses from all feature layers, while the inner summation accumulates information from K-sampled positions within each layer. $F_{l}\left(p_{k}+∆p_{k};c\right)$ is the feature value at the kth sampling position on the lth feature layer and channel c, where $∆p_{k}$ is the self-learned spatial offset. The learnable scalar $∆m_{k}$ measures the importance of the sampled position, and $w_{l,k}$ is the weight at the lth layer and the kth position, and $p_{k}$ is the coordinate of the kth position. The normalization coefficient 1/L ensures averaging across different feature levels.

(11) $$\pi_{C}\left(F\right)\cdot F=max\left(\alpha_{1}\left(F;\theta_{\alpha }\right)\cdot F_{c}+\beta_{1}\left(F;\theta_{\beta }\right),\alpha_{2}\left(F;\theta_{\alpha }\right)\cdot F_{c}+\beta_{2}\left(F;\theta_{\beta }\right)\right)$$

In Equation (11), $F_{c}$ represents the feature slice of the cth channel, $\alpha_{1}$ and $\alpha_{2}$ are channel activation weights, and $\beta_{1}$ and $\beta_{2}$ are the corresponding bias terms. $\alpha_{1},$ $\alpha_{2},$ $\beta_{1},$ and $\beta_{2}$ are generated by a hyper-function. The parameters $\theta_{\alpha }$ and $\theta_{\beta }$ are learnable and optimized during training via backpropagation, allowing the network to dynamically adjust channel responses for different tasks.

### 3.3. Experimental Environment and Model Hyperparameters

To ensure the authenticity and effectiveness of the experiment, the hyperparameters used in the experimental training process were kept consistent, shown in Table 1. These hyperparameters were determined through preliminary experiments to ensure fair comparison across different architectures. Specifically, the learning rate, weight decay, and momentum were chosen based on common practices in object detection tasks and validated through pilot experiments to ensure they provided reasonable performance for all models tested. In this experiment, the operating system is Windows 11. The CPU is Intel(R) Core (TM) i9-10900K and the GPU is dual NVIDIA RTX 3090. PyTorch 2.22 is used as the framework. The programming language is Python 3.8 and CUDA 12.1 is used for accelerated computing.

### 3.4. Model Evaluation Indicators

To evaluate the detection ability of the improved YOLOv8n model, we use F_1_-score, mAP@.50, and mAP@.50:.95 as the evaluation indicators. The *F*_1_-score, as a harmonic mean of accuracy and recall, can simultaneously reflect the accuracy and comprehensiveness of the model in identifying positive samples, providing a more robust performance evaluation. In addition, the two commonly used mean average precision (mAP) in object detection, i.e., mAP@.50 and mAP@.50:.95, are also used in this study. mAP@.50 refers to the average precision calculated at a threshold of 0.50, which is mainly used to evaluate the detection performance of the model under relatively relaxed conditions. The mAP@.50:.95, the averaged over the IoU thresholds from 0.50 to 0.95 with an interval of 0.05, provides a more rigorous and comprehensive performance evaluation and is stricter than mAP@.50. In practical applications, mAP@.50:.95 can better reflect the comprehensive ability and robustness of the model.

The formulas of the evaluation indicators of *F*_1_-score and *mAP* are expressed as follows:

(12) $$F_{1}=\frac{2PR}{P+R}$$

(13) $$mAP=\frac{1}{N}\sum_{t=1}^{N}AP\left(t\right)$$

In Equation (12), P=TP/(TP+FP)×100% and R=TP/(TP+FN)×100%. Here, P represents precision, and R represents recall. TP represents true positive, indicating the number of samples correctly predicted as positive by the model. FP represents false positive, indicating the number of samples that the model incorrectly predicts as positive. FN represents false negative, indicating the model incorrectly predicts the number of samples in the negative class. In Equation (13), $AP=\int_{0}^{1}P\left(R\right)dR,$ here $P\left(R\right)$ is the precision at the summon rate R and *N* is the number of thresholds.

## 4. Experiments and Results

### 4.1. YOLOv8 Models of Different Scales

The YOLOv8 models can be divided into YOLOv8n, YOLOv8s, YOLOv8m, YOLOv8l, and YOLOv8x based on their scales. These five models were trained on the same tomato dataset, and the training results are presented in Table 2. It shows that YOLOv8l and YOLOv8x with larger computation complexity theoretically have stronger feature extraction capabilities, but their actual performance has not reached the best. YOLOv8x has the highest parameters and GFLOPs (68.129 M and 257.4 G), but its mAP@.50 is only 78.1%. The parameters, GFLOPs and mAP@.50 of YOLOv8l, is 43.611 M, 164.8 G, and 77.1%, all of which are smaller than YOLOv8x. Compared with YOLOv8x, YOLOv8n has significantly reduced computational complexity but with the highest detection accuracy. This may be due to the fact that the dataset size is small with only 1000 images and the larger models (such as YOLOv8x and YOLOv8l) are prone to overfitting and causing lower performance. YOLOv8n has the advantages of simple structure, fast training speed, and low resource consumption, making it suitable for rapid prototyping and validation in this experiment. Therefore, YOLOv8n was chosen as the foundation model for subsequent model improvements.

### 4.2. Selection of Loss Function

To verify the effectiveness of the selected PIoU loss function, we conducted the comparative experiments of PIoU and other five loss functions for evaluation, including the v2 version of PIoU and four commonly used loss functions: CIoU, GIoU, DIoU, and EIoU. The results are shown in Table 3. The original YOLOv8n model uses CIoU as the loss function. Compared with it, the PIoU loss function we adopted improved mAP@.50 and mAP@.50:.95 by 1.1% and 0.3%, respectively, which shows that the PIoU loss function performs the best.

### 4.3. Effect of FADC Module on Feature Extraction and HSPAN on Feature Fusion

**Figure 8:**
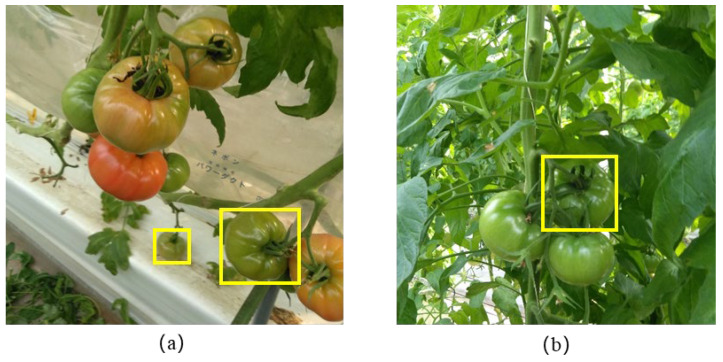
Tomatoes with different spatial positions (**a**) and in complex backgrounds (**b**).

In tomato variety and maturity detection, we mainly face two problems. On the one hand, the different locations of tomatoes of the same variety lead to significant differences in their feature information. On the other hand, in complex backgrounds, the feature of tomatoes needs to be more obvious. These two problems are shown in Figure 8. The two yellows boxes in Figure 8a show two tomato fruits with different locations. Figure 8b shows the example of the tomato fruits in the complex background (one of them was marked by the yellow box).

**Figure 9:**
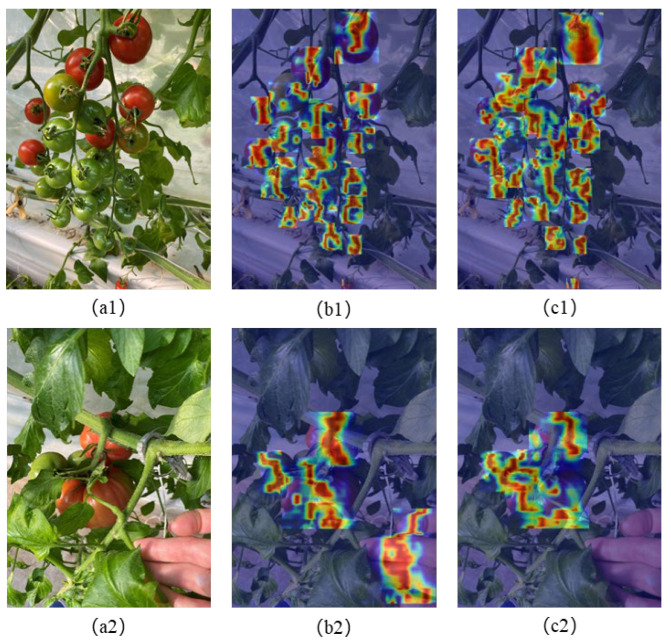
Heatmap comparison of YOLOv8n and our model on tomatoes in different scenarios: (**a1**–**c1**) multi-tomato scenario, (**a2**–**c2**) complex background, (**a1**,**a2**) original image, (**b1**,**b2**) heatmap of YOLOv8n, and (**c1**,**c2**) heatmap of our model.

In the proposed model, the FADC module performs local feature extraction to enable multi-level screening of feature information. To more intuitively show the detection effect of this model on tomato fruits with different maturity levels in complex backgrounds (with leaves, stem, or human hands, etc.), heatmaps generated by HiResCAM were used to highlight the areas that the model focuses on, as shown in Figure 9. The red color in the heatmaps means the high probability area where the model believes in the locations of tomatoes. It can be seen that the object area and feature information obtained by our model is more accurate. For example, our model identified the top big mature fruit and the small mature fruit at the lowest edge in Figure 9a1 more accurately than YOLOv8n. In addition, from Figure 9b2, it shows that YOLOv8n mistakenly identified a human hand as tomatoes on the heatmap, while our model distinguished it more accurately from the background. This shows that the FADC module can obtain feature information more accurately and effectively. At the same time, the HSPAN structure further focuses on crucial information, enhances the response to critical features of different maturity, and selectively fuses this information. This design enables the model to better capture the details of both small and large tomatoes, reduce the interference of background feature, and thus improve detection accuracy.

**Figure 10:**
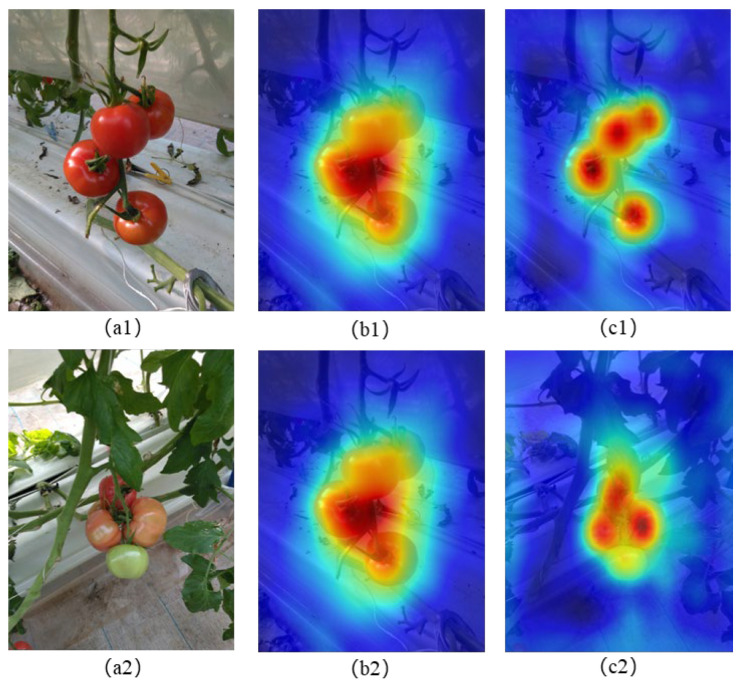
Visualization comparison of global perception ability: original images (**a1**,**a2**), and global perception maps of YOLOv8n (**b1**,**b2**), and of our model (**c1**,**c2**). Blue indicates background regions and red represents the interested regions of the models.

In addition to showing the local attention distribution of the model through the heatmap generated by HiResCAM, the global perception ability of the model is also studied. In order to visualize the global perception of the model, based on the feature activation visualization method [30], we extract the feature activation maps of different layers of FADC module and HSPAN structure, and generate a comprehensive heatmap through channel aggregation and multi-scale weighted fusion, which intuitively shows how the model perceives the targets in the global scope. Figure 10 shows the global perception visualization comparison of our model and YOLOv8n. It shows that our model has stronger global perception ability. For example, Figure 10c1 shows our model is more accurate in the extraction and fusion of different tomato contour features as well as the features of background leaves, while the YOLOv8n model is poor in overall perception. Figure 10c2 presents the detected tomatoes of different maturity levels with partial overlap (shown in Figure 10a2), from which it shows our model has the better global perception responses according to the different maturity characteristics of tomatoes and detailed morphology of background leaves. The superior global perception ability of our model shows the HSPAN structure in the neck part can well fuse features of different scales, and retain important information while discarding redundant details, thus effectively handling global features with complex scenarios (for example, different maturity levels and partial overlap).

### 4.4. Analysis of DyHead Detection

**Figure 11:**
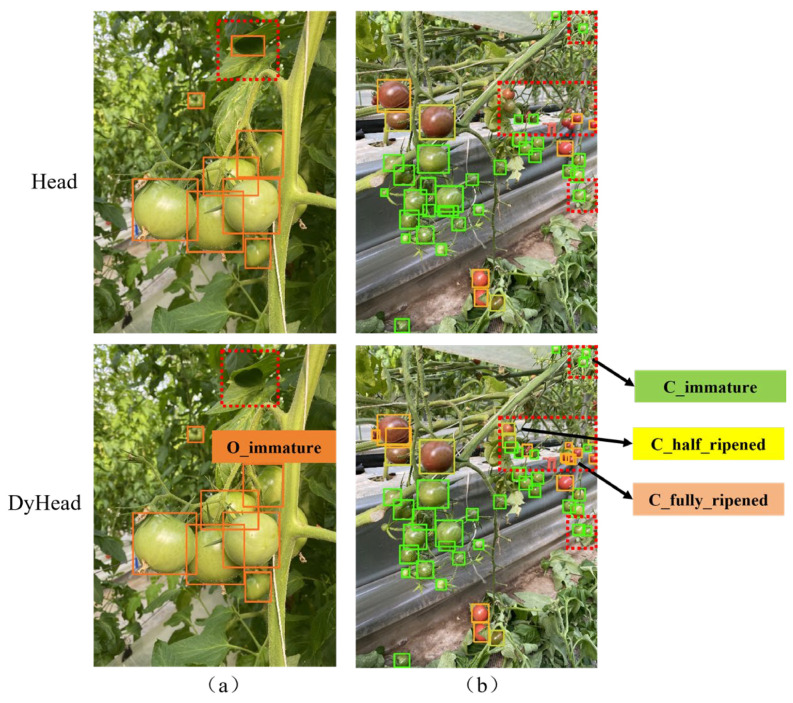
Detection results of ordinary tomatoes (**a**) and cherry tomatoes (**b**) by different detection heads. The color boxes indicate maturity levels: green for immature, yellow for fully ripe, and yellow-green for half-ripe.

To prove that the detection effect of DyHead is better, Figure 11 shows the detection results of the original detection head and DyHead on different tomatoes. The orange solid boxes in Figure 11a are for immature ordinary tomatoes and the three colors of boxes in Figure 11b are for cherry tomatoes (green for immaturity, yellow for fully ripe, and yellow green for half ripe). In Figure 11, dotted red boxes are for marking the mistaken or missed detection by original head but correctly detected by DyHead. For example, in Figure 11a, the shadow of a piece of leaf is detected as an unripe tomato by the original detection head. In contrast, DyHead with the spatial perception attention mechanism can effectively focus on the critical position and reduce the interference of background noise, especially in the case of light and shadow. Therefore, there is no false detection on this part when using DyHead. In addition, our model can also correctly classify tomatoes that were partially occluded. In Figure 11b, there are many tomatoes with different maturity levels and some of the tomatoes marked in red dotted boxes were missed by the original detection head but well detected by the Dyhead. It is due to the Dyhead enables the model to adaptively adjust the feature representation and the introduced task-aware mechanism to assign feature channels to specific tasks makes multiple classification tasks can be processed simultaneously. During training, the four coordinates of each bounding box serve as regression targets for the detection head, so the network simultaneously learns the bounding box size (width and height) and spatial position (center). These geometric features are then fused with color/texture cues inside the HSPAN neck and further processed by the dynamic detection head, which adaptively reweights channel and spatial responses according to local context. In this way, apparent size and location information are explicitly exploited to refine ripeness classification.

### 4.5. Ablation Experiments

In this study, the improvements include using HSPAN structure for feature selection and fusion as the neck part, replacing the original detection head with a dynamic detection head, and introducing the FADC module and PIoU loss. To verify the impact of these improvements on the model performance, we conducted ablation experiments under the same environment and hyperparameter settings. By systematically removing some components of the network model and evaluating their contribution to the overall performance, it shows the model design is optimized and the interpretability is improved with adding these components. In this section, we use the YOLOv8n model as the baseline and add different improvement modules. The ablation experiment results are shown in Table 4.

Table 4 is the comparison of our model with the baseline YOLOv8n. It shows that, the use of each individual module (FADC, HSPAN, DyHead) can improve the model performance (mAP@.50 = 0.808–0.816) compared with the baseline YOLOv8n (mAP@.50 = 0.804). The combination of two modules performs better detection performance (mAP@.50 = 0.818–0.821) than with only one module (mAP@.50 = 0.752–0.754). The more modules added, the better performance of the model. Specifically, adding FADC module improves the F_1_-score, mAP@.50, and mAP@.50:.95 by 0.6%, 0.4%, and 0.4%, and the HSPAN structure improves them by 0.7%, 1%, and 1.2%, respectively, which shows that these two modules slightly improve the model performance. The dynamic detection head improves them by 0.5%, 1.2%, and 1.7%, respectively, indicating that the adaptive adjustment for processing different tomato features can effectively utilize the features of different levels and scales. In tomato variety and maturity detection, this flexibility enables the detection to maintain high accuracy in different backgrounds.

Compared with the baseline YOLOv8n, adding the combination of FADC + HSPAN improves the above three indicators by 1.4%, 1.7%, and 1.8%, respectively. Further adding DyHead, i.e., FADC + HSPAN + DyHead, the F_1_-score and mAP@.50 are increased by 1.7% and 2% (without further improvement on mAP@.50:.95). If further adding PIoU, i.e., our model, the three indicators increased by 2.7%, 3.1%, and 2.1%, which shows that the PIoU loss function better adapts to the detection of different objects and improves the detection effect. Overall, compared with the baseline YOLOv8n, our model shows the best detection performance.

For the computational complexity in Table 4, it shows that the parameters and GFLOPs of YOLOv8n are 3.006 M and 8.1. The introduction of the HSPAN structure significantly reduces the parameters to 2.082 M and GFLOPs to 7.1, while the DyHead obviously increases the parameters to 3.486 M and GFLOPs to 9.6. Combining FADC and HSPAN causes 2.099 M parameters and 7.0 GFLOPs, maintaining a low computational footprint, indicating FADC does not obviously influence computational complexity. Our model with all modules (FADC, HSPAN, DyHead, and PIoU) has 2.841 M parameters and 8.9 GFLOPs, achieving the better balance between performance and computational efficiency.

### 4.6. Comparison Experiment

The performance of our model was verified through comparison with several lightweight models (YOLOv9t [31], YOLOv10n [32], YOLOv11n [33], and YOLOv12n [34]) and several mainstream models (Faster-RCNN [35], RT-DETR-r18 [36], and the baseline YOLOv8n). All these models were retrained from scratch and evaluated under the same experimental settings on the same platform and software version to ensure reproducibility and fair comparison.

The comparative experimental results are shown in Table 5. It shows that compared with the lightweight models of YOLOv9t, YOLOv10n, YOLOv11n, and YOLOv12n, mAP@.50 of our model increased by 6.1%, 9.6%, 4.8%, and 6.6%, and mAP@.50:.95 by 5.4%, 8.3%, 4.5%, and 7.6%, respectively. These results indicate that our model exhibits better performance in this task. This also indicates that the performance of a model is not solely determined by the version, but is closely related to the characteristics of the dataset used. Different datasets have different features and complexities, so the highest versions of a model may not achieve the best performance.

Compared with several mainstream models, our model also achieved the highest accuracy in both mAP@.50 and mAP@.50:.95. It is worth noting that although Faster-RCNN also performed well in these two indicators (0.813 and 0.646), even exceeding YOLOv8n, it has too many parameters and GFLOPs to cope with actual detection tasks. Among all of these models, our model has the highest accuracy (0.835) with lower parameters (2.841 M, only 0.81 times that of YOLOv8n), indicating our model achieves a better balance between performance and computational efficiency. The introduction of dynamic detection heads in our model increases the parameters and computation complexity, while the HSPAN network structure reduces the parameters by optimizing the original feature fusion process. Therefore, compared with the baseline YOLOv8n, our model reduces the parameters and increases GFLOPs. In addition, our model maintains a competitive inference speed, with an FPS of 165.4, which is comparable to other lightweight YOLO models. Overall, our model demonstrates superior detection accuracy, competitive inference speed, and balanced computational complexity, making it a highly efficient and practical solution for the simultaneous detection of tomato variety and maturity.

## 5. Conclusions

Based on the YOLOv8n model, this study proposes the simultaneous detection model of tomato variety and maturity with the improvements on accuracy and computation performance. Specifically, the C2f module in the backbone part is replaced by FADC, which can effectively expand the receptive field by dynamically adjusting the expansion rate of the convolution kernel while retaining detailed features, thereby better capturing local information and improving the detection accuracy. In addition, in the low-level and high-level feature fusion stage, we introduced the CA and the SFF module and proposed a path aggregation network structure with advanced filtering function to improve the detection performance. PIoU loss function is used to adaptively optimize the predicted bounding box. The dynamic detection head is introduced to perform different classification tasks to further improve the performance of the model in complex backgrounds.

This study shows that our model performs the best performance among those comparative models, with mAP@.50 being 83.5% higher than that of the baseline YOLOv8n (80.4%). In addition, the maturity detection of our model on apples and citrus of our model shows its good generalization on other fruits. For the next improvement of our model, increasing the backbone network capacity and sample quantity of datasets can further enhance its detection accuracy. This study provides a theoretical basis and a practical lightweight solution for automated tomato picking and can be readily extended to other horticultural crops.

## Figures and Tables

**Table 1:** Training hyperparameters.

Parameter	Value
Image Size	640 × 640
Learning Rate	0.01
Weight Delay	0.0005
Momentum	0.937
Optimizer	SGD
Batch Size	4
Epoch	150

**Table 2:** The comparison of different YOLOv8 models.

Name	mAP@.50	mAP@.50:.95	Parameters (M)	GFLOPs
YOLOv8n	0.804	0.645	3.006	8.1
YOLOv8s	0.748	0.646	11.127	28.4
YOLOv8m	0.765	0.606	25.843	78.7
YOLOv8l	0.771	0.639	43.611	164.8
YOLOv8x	0.781	0.655	68.129	257.4

**Table 3:** Comparison of different loss functions.

	Name	F_1_-Score	mAP@.50	mAP@.50:.95
YOLOv8n	+CIoU	0.764	0.824	0.663
	+GIoU	0.762	0.819	0.660
	+DIoU	0.749	0.818	0.657
	+EIoU	0.768	0.827	0.666
	+PIoUv2	0.759	0.816	0.652
	+PIoU	0.774	0.835	0.666

**Table 4:** Results of the ablation experiments.

	FADC	HSPAN	DyHead	PIoU	F_1_-Score	mAP@.50	mAP@.50:.95	Parameters (M)	GFLOPs
YOLOv8n					0.747	0.804	0.645	3.006	8.1
	√				0.753	0.808	0.649	3.024	8.0
		√			0.754	0.814	0.657	2.082	7.1
			√		0.752	0.816	0.662	3.486	9.6
	√	√			0.761	0.821	0.663	2.099	7.0
	√		√		0.760	0.819	0.662	3.508	9.6
		√	√		0.762	0.818	0.662	2.828	9.1
	√	√	√		0.764	0.824	0.663	2.841	8.9
Ours	√	√	√	√	0.774	0.835	0.666	2.841	8.9

**Table 5:** Results of the comparison experiments.

Name	mAP@.50	mAP@.50:.95	Parameters (M)	GFLOPs	FPS
YOLOv9t	0.774	0.613	1.971	7.6	185.2
YOLOv10n	0.739	0.584	2.696	8.2	173.9
YOLOv11n	0.787	0.622	2.583	6.3	198.4
YOLOv12n	0.769	0.591	2.509	5.8	207.6
Faster-RCNN	0.813	0.646	41.374	178.0	28.6
RT-DETR-r18	0.404	0.280	20.093	58.3	65.5
YOLOv8n	0.804	0.645	3.006	8.1	177.7
**Ours**	0.835	0.667	2.841	8.9	165.4

## Data Availability

The original contributions presented in this study are included in the article. Further inquiries can be directed to the corresponding author.
